# Predictors and Correlates of Depression and Anxiety Symptom Trajectories in a Large Digital Mental Health Provider: Retrospective Analysis of Data From Rula Health

**DOI:** 10.2196/75750

**Published:** 2025-07-25

**Authors:** Kelsey Lynn McAlister, Lara Baez, Douglas Newton, Sam Seiniger, Amy Pearlman, Allie Woodhouse, Jennifer Huberty

**Affiliations:** 1 Fit Minded Inc, 2901 E Greenway Road PO Box 30271, Phoenix, AZ, 85046, United States, 1 (602) 935-6986; 2 Rula Health, Santa Clara, CA, United States

**Keywords:** measurement-based care, teletherapy, web-based therapy, digital mental health interventions, mental health, clinical outcomes, real-world evidence

## Abstract

**Background:**

Depression and anxiety are highly prevalent and burdensome, yet many individuals, especially those with subclinical symptoms, remain underserved by traditional care models. While digital mental health interventions (DMHIs) have improved access, few integrate high-frequency measurement-based care (MBC) or evaluate outcomes across the full spectrum of symptom severity in real-world settings.

**Objective:**

The purpose of this study was to examine the effects of participation in a commercial MBC DMHI, Rula Health, on changes in depression and anxiety over time in both subclinical and clinical patients. We aimed to (1) explore the trajectories of anxiety and depression symptoms and (2) examine the impact of demographics and primary diagnosis on depression and anxiety trajectories.

**Methods:**

We retrospectively analyzed longitudinal symptom data from adults receiving therapy through Rula Health, an MBC-based DMHI. Depression, via the Patient Health Questionnaire-9 (PHQ-9), and anxiety symptoms, via the Generalized Anxiety Disorder-7 (GAD-7), were measured before each visit over 12 therapy visits. Linear spline mixed-effects models with a knot placed at 5 visits (based on visual inspection) were used to evaluate symptom trajectories and identify moderators of treatment response, including demographic characteristics and primary diagnosis.

**Results:**

A total of 365,741 adults (mean age 37.03, SD 11.81 years; 238,839/360,388, 66.27% female; 87,758/145,947, 60.13% White) with 2,685,103 therapy visits were included in the sample. Baseline depression (ie, PHQ-9) and anxiety (ie, GAD-7) scores averaged 9.41 (SD 6.61) and 9.45 (SD 5.65), respectively, decreasing to 6.37 (SD 5.83) and 6.50 (SD 5.01) within 12 visits. Depression (β=−0.72, *P*<.001) and anxiety (β=−0.72, *P*<.001) symptoms decreased significantly over the first 5 visits, and to a lesser degree over visits 6‐12 (depression: β=−0.02, *P*<.001; anxiety: β=−0.0078, *P*=.004). Faster improvements over visits 1‐5 occurred in younger patients (β_PHQ_=0.0031, *P*<.001; β_GAD_=0.0027, *P*<.001), and those identifying as Black/African American (β_PHQ_=–0.089, *P*<.001; β_GAD_ =−0.042, *P*<.001), American Indian/Alaska Native (β_PHQ_=–0.14, *P*<.001; β_GAD_=−0.11, *P*<.001), and Native Hawaiian/Pacific Islander (β_PHQ_=−0.12, *P*<.001; β_GAD_=−0.069, *P*=.01). Patients with an anxiety (β_GAD_=−0.071, *P*<.001) or trauma-related (β_GAD_=−0.021, *P*=.03) disorder had faster improvements in GAD-7.

**Conclusions:**

This study contributes to the growing evidence base from commercial DMHIs by demonstrating significant improvements in depression and anxiety symptoms across both clinical and subclinical populations using real-world data from a large, national provider. Symptom reductions were most rapid in the first 5 visits, with continued improvements through session 12, especially among historically underserved groups. These findings highlight Rula Health’s ability to deliver early, sustained, and equitable outcomes through an MBC model.

## Introduction

Depression and anxiety are prevalent mental health disorders in the United States, characterized by persistent sadness and loss of interest and excessive worry, respectively [1,2]. National estimates indicate that 39% of adults experience depressive symptoms, while 42% report anxiety symptoms, highlighting the widespread nature of these conditions [2]. A significant proportion of individuals report subclinical symptoms (ie, lower severity symptoms on standard measures of depression and anxiety), yet still experience functional impairment and are at elevated risk of developing clinical disorders [3,4]. The economic burden of these disorders is equally significant—major depressive disorder alone accounts for $92.7 billion in annual costs, nearly half of which is attributed to treatment-resistant depression [5]. Health care expenditures for individuals with anxiety disorders average $7906 per person annually, with costs rising substantially when anxiety coexists with other chronic conditions [6]. Compounding their impact, depression and anxiety frequently co-occur, further impairing emotional regulation, cognitive function, and interpersonal relationships [7,8]. Given their pervasive nature and considerable societal costs, effective interventions are crucial.

Significant gaps remain in the effective treatment of depression and anxiety. Traditional mental health care models, which rely on in-person therapy and pharmacological interventions, often fail to meet demand due to limited provider availability, high costs, and logistical challenges [9]. These limitations are particularly relevant for subclinical individuals with less severe symptoms, who face barriers to care such as stigma, low perceived need, and lack of tailored treatment options [10,11]. Digital mental health interventions (DMHIs), including web-based therapy, have emerged as a solution to bridge the gaps in accessibility, affordability, and convenience, offering individuals an alternative way to connect with licensed therapists and mental health resources. While DMHIs have shown promise in expanding access to mental health support [12], questions remain about their effectiveness and integration into existing, real-world care models. Evidence suggests that digital therapy is just as effective as in-person therapy in improving clinical outcomes, but most studies are randomized controlled trials with limited real-world generalizability [13-15]. Furthermore, many therapist-guided DMHIs rely on standardized, one-size-fits-all treatment approaches that fail to adapt to the unique and evolving needs of users [16]. Without personalized treatment adjustments or real-time symptom tracking, patients may struggle to remain engaged, and clinicians may lack the necessary insights to optimize care delivery.

Measurement-based care (MBC) has emerged as a promising approach to improving mental health treatment by systematically tracking symptoms and using real-time data to guide personalized care [17]. Despite its well-documented benefits [18], adoption remains low—fewer than 20% of mental health practitioners use it consistently, and only 5% implement it at the recommended frequency [19]. Although barriers such as lack of standardization, limited clinical integration, and inconsistent patient engagement hinder the effectiveness of MBC in DMHIs, integrating MBC effectively could tailor treatment and optimize therapy dosage to improve clinical outcomes.

Despite the potential of MBC-based therapy DMHIs, key challenges remain in its implementation and effectiveness. While research supports the effectiveness of teletherapy for depression and anxiety [20-22], most studies do not use large, representative patient samples from real-world DMHIs and do not evaluate interventions that use symptom data in real time to guide clinical care, as is characteristic of true MBC. DMHI studies often focus on individuals with moderate to severe symptoms [23,24], despite evidence that those with lower severity symptoms are at high risk of developing clinical disorders [3,4]. This emphasis may lead to the underrepresentation of those with subclinical presentations, potentially affecting the applicability of the findings to the general population. Additionally, subclinical samples are 2-3 times more likely to end up with clinical symptoms, contributing to higher long-term societal costs such as increased health care use and reduced economic productivity [3,4,25]. Furthermore, little is known about how treatment response evolves over time within commercial DMHIs. While nonlinear change in therapy is well-established in the broader literature [26], understanding longitudinal symptom change in commercial DMHIs is critical for optimizing care decisions, preventing premature dropout or overtreatment, and ensuring that MBC tools function effectively in large-scale, real-world contexts. Additionally, while a meta-analysis of randomized controlled trials found that interventions lasting at least 7 weeks significantly reduced anxiety symptoms, effects on depression were inconsistent [27]. This suggests that treatment duration may not fully account for symptom improvement, and other factors, such as demographic and clinical characteristics, may influence these outcomes. Given these gaps, further research is needed to evaluate the effectiveness of commercial MBC-integrated DMHIs, such as examining how symptoms change over time and identifying key factors that shape symptom response.

The purpose of this study was to examine the effects of participation in a commercial MBC DMHI, Rula Health, on changes in depression and anxiety over time in both subclinical and clinical patients. We aimed to (1) explore the trajectories of anxiety and depression symptoms and (2) examine the impact of demographics and primary diagnosis on depression and anxiety trajectories.

## Methods

### Study Design and Participants

This retrospective study analyzes secondary data from Rula Health, a DMHI that connects individuals with licensed therapists and psychiatric providers across the United States. Rula Health’s treatment model is grounded in MBC and personalized treatment to enhance clinical outcomes. Individuals were eligible for inclusion if they were adults aged 18 years or older and received therapy treatment through Rula Health between August 20, 2020, and March 31, 2025. Only therapy visits were included for analysis. Eligible participants were identified using existing clinical records collected in the course of routine care (ie, retrospective cohort sampling). Because the study was retrospective, no direct recruitment of participants occurred.

### Ethical Considerations

All patients at Rula Health complete an informed consent to care prior to treatment, which outlines the scope of services, confidentiality policies, and data usage information. This consent process is separate from and in addition to agreeing to Rula Health’s privacy policy, which outlines how their data may be used and protected. This study involves a retrospective analysis of deidentified secondary data, qualifying it for exemption from additional consent requirements under human participant research regulations. The Biomedical Research Alliance of New York (BRANY) Institutional Review Board (Study ID 25-035-2061) approved this study. Participants did not receive compensation for this study.

### Treatment

Patients learn about Rula Health through various referral sources, including primary care providers, insurance providers, employer-sponsored mental health benefits, health care partners, and direct online searches. The platform is designed to simplify access to mental health care while ensuring that patients have control over their provider selection. After completing a brief intake questionnaire, individuals are matched with a curated list of licensed therapists or psychiatric providers based on their mental health concerns, insurance coverage, and personal preferences, such as provider specialty, background, and availability. Rather than being assigned a provider, patients can choose from these matches, allowing them to select someone who best aligns with their needs and preferences.

Once matched, patients begin treatment through secure video sessions with their chosen provider. During the initial session, the provider collaborates with the patient to develop a personalized treatment plan that outlines therapy goals, intervention strategies, and expected progress. Rula Health’s care approach integrates MBC, meaning that patients complete standardized symptom assessments at regular intervals. These assessments help track changes in symptom severity and inform data-driven adjustments to treatment plans. Additionally, Rula Health’s model emphasizes provider quality, equipping clinicians with tools and insights that support evidence-based care while monitoring treatment effectiveness to ensure high standards of service.

Therapy sessions are scheduled based on individual patient needs, with some patients engaging in weekly sessions and others biweekly or a more flexible schedule, depending on symptom severity, treatment goals, and provider recommendations. Rula Health providers use a variety of evidence-based therapeutic approaches, including cognitive behavioral therapy, dialectical behavior therapy, acceptance and commitment therapy, and other empirically supported interventions tailored to each patient’s needs. The treatment process is collaborative, with therapists working closely with patients to establish personalized goals, track progress, and adjust interventions as needed. Patients complete assessments on their mental health symptoms at regular intervals, and Rula Health encourages providers to use these assessment scores to guide the patient’s treatment as part of MBC.

### Study Measures

Upon intake, patients report demographic information including age, biological sex, race, and ethnicity. Providers report the patient’s primary diagnosis. Among patients with completed assessments, mental health symptom assessments were completed before each scheduled appointment or up to once per week starting August 20, 2020. Starting December 8, 2024, patients are asked to complete these assessments every 2 weeks. This analysis incorporates assessment data on depression and anxiety symptoms.

### Depression and Anxiety Measures

The Patient Health Questionnaire-9 (PHQ-9) is a 9-item tool that assesses the severity of depressive symptoms over the past 2 weeks. Patients rate each item on a 4-point Likert scale from 0 (“Not at all”) to 3 (“Nearly every day”). The total score ranges from 0 to 27. The PHQ-9 has demonstrated high internal consistency, with a Cronbach α of .89, and excellent test-retest reliability, with a correlation coefficient of 0.84 [28]. Higher scores indicate greater depression severity [29].

The Generalized Anxiety Disorder-7 (GAD-7) is a 7-item self-report questionnaire that evaluates the severity of anxiety symptoms over the past 2 weeks. Patients rate each item on a 4-point Likert scale, ranging from 0 (“Not at all”) to 3 (“Nearly every day”), with total scores spanning from 0 to 21. The GAD-7 is widely used and validated, demonstrating high internal consistency (Cronbach α=.89‐0.92), strong test-retest reliability (intraclass correlation=0.83) [30,31]. Higher scores indicate greater anxiety severity [32,33].

### Statistical Analyses

Data were limited to the first 12 visits because this is a common length of treatment in clinical trials [34], and thus estimates a relative episode of care at Rula Health. If outcomes data were missing for the 12th visit, or if the patient was not in treatment for a full 12 visits, data from the nearest visit prior to the 12th visit was used to calculate all change metrics. Because of the very large number of primary diagnoses given by providers, patient diagnoses were collapsed into 4 categories based on *Diagnostic and Statistical Manual of Mental Disorders, Fifth Edition*, categories: Anxiety Disorders, Depressive Disorders, Trauma and Stress-Related Disorders, and Other Disorders (the “Other” category captured any diagnosis not in the 3 categories). The primary diagnosis was reported for each visit; thus, a person-level variable representing each individual’s modal diagnostic category was calculated. The modal diagnostic category was used in all subsequent analyses. Demographic characteristics and descriptive statistics were calculated and reported for the sample. Cohen *d* was calculated to estimate the effect size of the change in depression and anxiety between baseline and up to 12 visits, with missing data handled using the last observation carried forward method. Due to high missingness in data at exactly 12 visits (~80%), sensitivity analyses were conducted to test for differences in PHQ-9 and GAD-7 change scores between patients with and without scores at 12 visits (see Multimedia Appendix 1). Given the difference observed between those with and without scores at 12 visits, multiple imputation was used to impute visit 12 PHQ-9 and GAD-7 scores to reduce bias and preserve statistical power in our findings. Because results were highly similar to the last observation carried forward analyses, these results are in the supplement.

**Figure 1.**
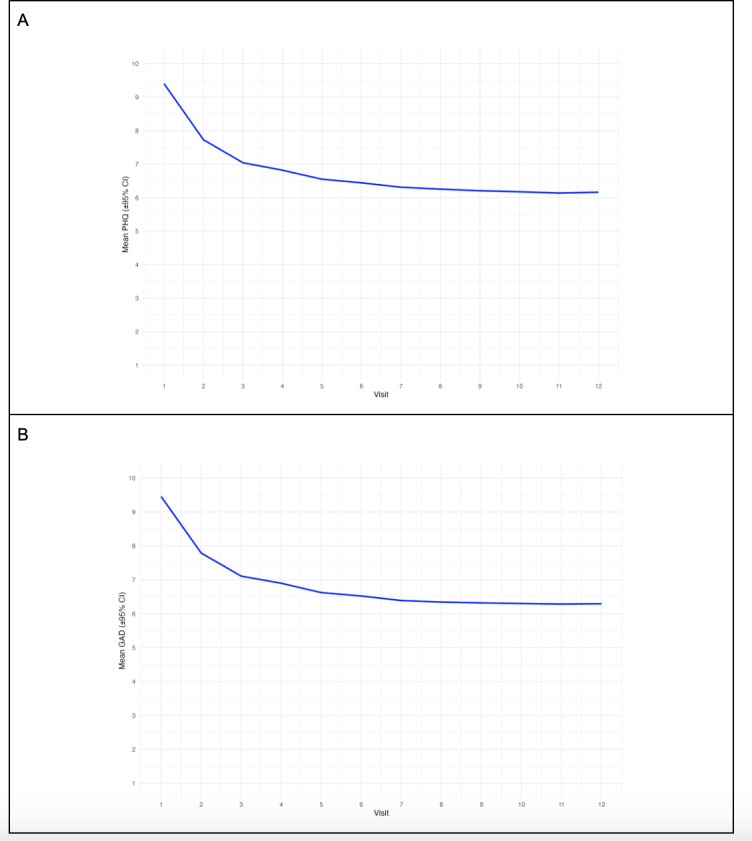
(A) Raw mean Patient Health Questionnaire-9 (PHQ-9) and (B) Generalized Anxiety Disorder-7 (GAD-7) score trajectories over 12 visits. 95% CIs were estimated but were too thin to visualize and report.

Linear mixed models were used to model changes in depression and anxiety symptoms over time in treatment at Rula Health. Visual inspection of the raw data indicated that a non-linear function of time should be applied (see Figure 1). Visual fit has been cited as a way to determine knot placement in spline models [35-38]. As a result, we applied a linear spline model with a knot at visit 5, allowing us to model slopes between visits 1‐5 and 6‐12 separately. A base model with random intercepts was estimated for depression and anxiety outcomes separately, then demographic covariates including gender, age, race, ethnicity, and modal primary diagnosis were added. Interactions between demographic variables and the slopes between 1‐5 visits and 6‐12 visits were added to test whether treatment effects differed based on demographic characteristics. Separate models were estimated for each demographic variable to simplify the interpretation of the interaction terms. Satterthwaite’s method was used to approximate the degrees of freedom and statistical significance of the slopes in the models. Models with random slopes were tested but did not converge and thus were not used. Restricted maximum likelihood estimation was used in all models. All statistical analyses were conducted in R (version 4.3.1; R Foundation for Statistical Computing) [39].

## Results

### Data Availability and Demographics

A total of 365,741 adults and 2,685,103 therapy visits were included in the analysis. Patients had a mean age of 37.03 (SD 11.81) years and were mostly female (238,839/360,388, 66.27%), Caucasian/White (87,758/145,947, 60.13%), and non-Hispanic (115,033/168,633, 68.21%). Patients’ modal primary diagnoses were anxiety disorder (116,754/350,495, 33.31%), depressive disorder (82,833/350,495, 23.63%), and trauma and stress-related disorder (107,968/350,495, 30.80%) (Table 1). Patients attended an average of 7.34 (SD 4.33) sessions and were in treatment for an average of 80.36 (SD 95.14) days. On average, patients reported baseline depression and anxiety scores on the border between mild and moderate symptoms (mean baseline depression=9.41, SD 6.61; mean baseline anxiety=9.45, SD 5.65), and final scores within 12 visits decreased to an average of 6.37 (SD 5.83) for depression and 6.50 (SD 5.01) for anxiety symptoms (Table 2). Effect sizes (ie, Cohen *d*) for change from baseline to up to 12 visits were moderate for depression (−0.54, 95% CI −0.55 to −0.54) and anxiety (−0.58, 95% CI −0.58 to −0.57).

**Table 1.** Descriptive statistics of categorical variables (=365,741).

Categorical variable	Value, n (%)
Gender (n=360,388 available)	
Female	238,839 (66.27)
Male	121,549 (33.72)
Race (n=145,947 available)	
African American/Black	17,834 (12.22)
American Indian/Alaska Native	1405 (0.96)
Asian	16,178 (11.08)
Caucasian/White	87,758 (60.13)
Mixed race	20,335 (13.93)
Native Hawaiian/Pacific Islander	2437 (1.67)
Ethnicity (n=168,633 available)	
Non-Hispanic	115,033 (68.21)
Hispanic	53,600 (31.79)
Primary diagnosis (n=350,495 available)	
Anxiety disorder	116,754 (33.31)
Depressive disorder	82,833 (23.63)
Trauma and stress-related disorder	107,968 (30.80)
Other	42,940 (12.25)

**Table 2.** Descriptive statistics of continuous variables (N=365,741).

Continuous variable	Data available, n (%)	Mean (SD)
Age	365,740 (100)	37.03 (11.81)
Days in treatment	365,741 (100)	80.36 (95.14)
Visits attended up to 12	365,741 (100)	7.34 (4.33)
Baseline depression symptoms	228,697 (62.53)	9.41 (6.61)
Last visit up to 12 depression symptoms	198,812 (54.36)	6.37 (5.83)
Change in depression symptoms	163,248 (44.63)	–3.14 (5.78)
Baseline anxiety symptoms	230,739 (63.09)	9.45 (5.65)
Last visit up to 12 anxiety symptoms	201,081 (54.98)	6.50 (5.01)
Change in anxiety symptoms	165,618 (45.28)	–3.03 (5.24)

### Patient Symptom Trajectories Over Time

Raw mean PHQ-9 and GAD-7 score trajectories over 12 visits are presented in Figure 1. Linear mixed effects model results showed that depression symptoms decreased significantly over the first 5 visits (β=–0.74, *P*<.001), and to a lesser degree over visits 6‐12 (β=−0.032, *P*<.001). There was a moderate negative correlation between fixed effect intercept and visit 1‐5 slope (*r*=−0.56), suggesting that people who start with higher depression symptoms decrease more quickly over the first 5 visits. There was also a small positive correlation between fixed effect intercept and visit 6‐12 slope (*r*=0.19), indicating that people who start with higher depression decrease more slowly over visits 6‐12.

The main findings were consistent when main effects of demographic covariates were added to the model. Furthermore, male gender (β=−0.72, *P*<.001), greater age (β=−0.039, *P*<.001), African American/Black race (β=−0.62, *P*<.001), and non-Hispanic ethnicity (β=−0.09, *P*<.001) were associated with lower depression symptom scores. American Indian/Alaska Native (β=0.77, *P*<.001), Native Hawaiian/Pacific Islander (β=0.87, *P*<.001), and mixed-race (β=0.37, *P*<.001) patients had more severe depression symptoms. Primary diagnosis of an anxiety disorder (β=−2.81, *P*<.001), trauma and stress-related disorder (β=−2.97, *P*<.001), or other disorder (β=−2.12, *P*<.001) was associated with lower depression symptom scores than primary diagnosis of a depressive disorder (Table 3).

Linear mixed effects model results showed that anxiety symptom scores decreased significantly over the first 5 visits (β=−0.72, *P*<.001), and to a lesser degree over visits 6‐12 (β=−0.021, *P*<.001). There was a moderate negative correlation between fixed effect intercept and visit 1‐5 slope (*r*=−0.59), suggesting that people who start with higher anxiety decrease more quickly over the first 5 visits. There was also a small positive correlation between fixed effect intercept and visit 6‐12 slope (*r*=0.20), indicating that people who start with higher anxiety decrease less quickly over visits 6‐12, although this effect is smaller than the correlation for visits 1‐5.

Main findings generally remained consistent when main effects of demographic characteristics were added to the model. The slope for visits 6‐12 was much smaller after accounting for covariates, suggesting that much of the variance in anxiety scores over visits 6‐12 was explained by demographic and diagnosis-related factors. Furthermore, male gender (β=−0.68 *P*<.001), increased age (β=−0.030, *P*<.001), and Black/African American race (β=−0.78, *P*<.001) were associated with lower anxiety symptom scores. American Indian/Alaska Native race (β=0.51, *P*<.001), and Native Hawaiian/Pacific Islander race (β=0.71, *P*<.001) were associated with greater anxiety symptom scores. Modal primary diagnoses of an anxiety disorder (β=−0.57, *P*<.001), trauma and stress disorder (β=−1.41, *P*<.001), and other disorder (β=−1.02, *P*<.001) were associated with lower anxiety symptom scores (Table 4).

**Table 3.** Multilevel model predicting depression symptom scores (model with all predictor simple effects) (n=135,307 patients over n=542,835 visits).

Predictor	β	SE	*t* test (*df*)	*P* value
Fixed effects				
Intercept	12.02	0.052	229.0044 (116,741.812)	<.001
Visits 1‐5	−0.72	0.0038	−184.94 (452,297.22)	<.001
Visits 6‐12	−0.02	0.0028	−6.74 (422,775.18)	<.001
Gender (male)	−0.55	0.031	−17.64 (131,069.97)	<.001
Age	−0.039	0.0012	−31.73 (129,838.83)	<.001
Race (reference: White)				
Black/African American	−0.62	0.046	−13.42 (133,106.95)	<.001
American Indian/Alaska Native	0.77	0.15	5.18 (128,333.92)	<.001
Asian	0.048	0.048	1.00 (129,399.30)	.32
Mixed race	0.37	0.047	7.88 (127,970.91)	<.001
Native Hawaiian/Pacific Islander	0.87	0.11	7.78 (126,938.01)	<.001
Ethnicity (non-Hispanic)	−0.09	0.040	−2.26 (128,476.59)	.02
Diagnosis (reference: depressive disorder)				
Anxiety disorder	−2.81	0.039	−71.37 (127,812.44)	<.001
Trauma and stress-related disorders	−2.97	0.040	−40.49 (128,965.93)	<.001
Other	−2.12	0.052	−74.39 (132,234.27)	<.001
Random effects				
Intercept	23.33	4.83	—^a^	—
Residual	11.47	3.39	—	—

a Not applicable.

**Table 4.** Multilevel model predicting anxiety symptom score (model with all predictor simple effects; 135,723 patients over 547,225 visits).

Predictor	β	SE	*t* test (*df*)	*P* value
Fixed effects				
Intercept	10.77	0.041	261.69 (141,149.02)	<.001
Visits 1‐5	−0.72	0.0036	−199.72 (461,512.29)	<.001
Visits 6‐12	−0.0078	0.0026	−2.85 (429,367.32)	.004
Gender (male)	−0.68	0.026	−25.50 (130,019.23)	<.001
Age	−0.030	0.0010	−28.44 (128,669.89)	<.001
Race (reference: Caucasian/White)				
Black/African American	−0.78	0.040	−19.57 (132,262.04)	<.001
American Indian/Alaska Native	0.51	0.13	3.98 (126,742.02)	<.001
Asian	−0.047	0.041	−1.15 (128,133.023)	.25
Mixed race	0.19	0.041	4.55 (126,566.28)	<.001
Native Hawaiian/Pacific Islander	0.71	0.096	7.36 (125,319.15)	<.001
Ethnicity (non-Hispanic)	−0.042	0.034	−1.21 (127,100.13)	.23
Diagnosis (reference: depressive disorder)				
Anxiety disorder	−0.57	0.034	−16.97 (126,382.77)	<.001
Trauma and stress-related disorders	−1.41	0.034	−41.06 (127,663.72)	<.001
Other	−1.02	0.045	−22.62 (131,359.02)	<.001
Random effects				
Intercept	16.81	4.10	—^a^	—
Residual	9.99	3.16	—	—

a Not applicable.

### Interaction Effects of Patient Characteristics on Symptom Trajectories Over Time

Results of the models, including interactions between demographic variables and the slopes between visits 1‐5 and visits 6‐12 for both depression and anxiety outcomes, are displayed in Table 5. Only interaction effects are displayed for simplicity. Results revealed that greater age was associated with slower improvement in both depression and anxiety over visits 1‐5. Black/African American race, American Indian/Alaska Native race, and Native Hawaiian/Pacific Islander race were associated with faster improvement in both depression and anxiety over visits 1‐5. Mixed race was associated with faster improvement in depression symptom scores over visits 1‐5, but not in the anxiety. Asian race was associated with slower improvement in anxiety symptoms over visits 1‐5. Non-Hispanic ethnicity was associated with slower improvement in both depression and anxiety over visits 1‐5. A primary diagnosis of an anxiety disorder was associated with slower improvement in depression but faster improvement in anxiety over visits 1‐5. A primary diagnosis of a trauma and stress-related disorder was associated with slower improvement in depression and anxiety, but to a lesser degree for anxiety symptoms. A primary diagnosis of other disorders was associated with slower improvement in both depression and anxiety over visits 1‐5.

Overall, effects for visits 6‐12 were much smaller than those for visits 1‐5. Male gender was associated with a greater decrease in both depression and anxiety symptoms over visits 6‐12. American Indian/Alaska Native race, Asian race, and mixed race were associated with faster improvements in both depression and anxiety over visits 6‐12. Non-Hispanic ethnicity was associated with slower improvements in both depression and anxiety over visits 6‐12. Primary diagnoses of anxiety disorder, trauma and stress disorder, and other disorders were associated with slower improvements in both depression and anxiety over visits 6‐12 compared to a diagnosis of a depressive disorder.

**Table 5.** Interaction models examining demographic and primary diagnosis moderation of depression and anxiety symptom change

Visits and interactions	Depression symptoms (135,307 patients over 542,835 visits)			Anxiety symptoms (135,723 patients over 547,225 visits)		
	β	SE	*P* value	β	SE	*P* value
Visit 1-5 slope						
Gender (male)	−0.014	0.0084	.09	−0.012	0.0078	.11
Age	0.0031	0.00032	<.001	0.0027	0.00030	<.001
Race (reference: White)						
Black/African American	−0.089	0.013	<.001	−0.042	0.012	<.001
American Indian/Alaska Native	−0.14	0.039	<.001	−0.11	0.036	<.001
Asian	0.012	0.012	.32	0.042	0.012	<.001
Mixed Race	−0.046	0.011	<.001	0.0099	0.010	.34
Native Hawaiian/Pacific Islander	−0.12	0.029	<.001	−0.069	0.027	.011
Ethnicity (non-Hispanic)	0.097	0.0090	<.001	0.063	0.0084	<.001
Diagnosis (reference: depressive disorder)						
Anxiety disorder	0.25	0.010	<.001	−0.071	0.0096	<.001
Trauma and stress-related disorder	0.23	0.011	<.001	0.021	0.0099	.03
Other	0.25	0.014	<.001	0.086	0.013	<.001
Visit 6-12 slope						
Gender (male)	−0.022	0.0064	<.001	−0.018	0.0060	.003
Age	−0.000099	0.00024	.68	0.000044	0.00023	.85
Race (reference: White)						
Black/African American	0.011	0.010	.27	0.020	0.0095	.03
American Indian/Alaska Native	−0.060	0.029	.04	−0.063	0.027	.02
Asian	−0.030	0.0093	<.001	−0.019	0.0087	.03
Mixed race	−0.013	0.0084	.11	−0.018	0.0079	.02
Native Hawaiian/Pacific Islander	−0.036	0.021	.09	−0.022	0.020	.26
Ethnicity (non-Hispanic)	0.036	0.0068	<.001	0.030	0.0064	<.001
Diagnosis (reference: depressive disorder)						
Anxiety disorder	0.080	0.0077	<.001	0.041	0.0071	<.001
Trauma and stress-related disorder	0.066	0.0080	<.001	0.045	0.0075	<.001
Other	0.093	0.011	<.001	0.066	0.010	<.001

## Discussion

### Principal Findings

The purpose of this study was to examine the effects of participation in a commercial MBC DMHI, Rula Health, on changes in depression and anxiety over time in both subclinical and clinical patients. We aimed to (1) explore the trajectories of anxiety and depression symptoms and (2) examine the impact of demographics and primary diagnosis on depression and anxiety trajectories. We found that depression and anxiety scores significantly improved over time, with greater improvements in scores over the first 5 visits and slower but continued improvements over visits 6‐12. Greater symptom severity at baseline was associated with greater initial improvements, but this effect diminished after 5 visits. Demographic factors influenced symptom trajectories, with Asian individuals and non-Hispanic individuals experiencing slower improvement. In contrast, younger Black/African American, American Indian/Alaska Native, Native Hawaiian/Pacific Island, and mixed-race individuals experienced faster symptom improvements, especially in visits 1‐5. A primary diagnosis of anxiety or trauma-related disorders was related to slower improvements in depression symptoms but faster improvements in anxiety symptoms compared to those with a primary diagnosis of depression, whereas other diagnoses were associated with slower progress across both measures. Male gender and certain racial groups showed greater symptom reductions during visits 6‐12.

This study represents one of the few evaluations of a commercially available MBC DMHI demonstrating reductions in depression and anxiety symptoms using real-world data from both subclinical and clinical patients in a large sample. We examined over 360,000 patients who completed up to 12 sessions over an average of 81 days, resulting in over 2.5 million therapy visits. Other studies leveraging real-life patient data from digital or in-person practices have less than 1% of the sample size reported here, and none of these report findings in both subclinical and clinical patients [40,41]. Additionally, commercial DMHIs often restrict their samples to clinically significant cases to boost observed treatment effects or meet insurance reimbursement criteria [40,42,43], overlooking a large population experiencing early or mild symptoms who are at heightened risk of progressing to clinical disorders if left untreated. For example, individuals with subclinical anxiety and depression are at increased risk for developing clinical disorders by 2.5 and 3 times, respectively [3,4]. A meta-analysis shows that interventions targeting subclinical populations are among the most cost-effective strategies for reducing depression and anxiety [25]. This inclusive approach enhances the generalizability and clinical relevance of the findings and may have implications for the economic value of scalable DMHIs. More research is needed on the cost-effectiveness of MBC-based DMHIs. Overall, we found depression and anxiety decreased within 12 treatment visits across all patients and the full spectrum of severity. To our knowledge, no other commercial MBC DMHIs have reported comparable outcomes.

Symptoms of depression and anxiety decreased markedly over the first 5 visits of care at the MBC DMHI, and to a lesser degree over visits 6‐12. After experiencing a steep decline in symptoms early in care, Rula Health patients appear to enter a steady growth phase, where their initial progress stabilizes, and they continue improving at a more gradual pace while reducing the risk of setbacks [44,45]. Similar patterns of early symptom reductions have been observed in other studies of DMHIs [46-50], aligning with the idea of “sudden gains” in therapy, where symptoms improve quickly at first, then level out [26]. Notably, these patterns were observed in a sample that included both subclinical and clinical individuals, suggesting that DMHIs like Rula Health may be effective across a broad spectrum of symptom severity. While early improvements may reflect true therapeutic gains, they may also be partially influenced by regression to the mean—a statistical phenomenon in which individuals with extreme baseline scores naturally tend to show improvement over time, independent of treatment effects—which may have contributed to our findings [51]. Given that sudden gains are associated with better short- and long-term outcomes [52,53], future research should explore factors that drive early symptom improvements, such as provider quality and expertise [52,54].

Younger age was associated with more rapid improvements in depression symptoms compared to older individuals. Older adults may experience a slower response to psychotherapy due to factors such as longer illness duration, differences in cognitive flexibility, or variations in engagement with DMHIs [55]. Gender differences also emerged, with male patients showing greater symptom reductions later in treatment (visits 6‐12). Prior studies suggest that men often delay help-seeking behaviors and may require more time in treatment, as they are less likely to disclose emotional distress and engage with therapy [56]. In contrast, women tend to show earlier symptom reductions, likely due to higher baseline engagement, greater willingness to disclose distress, and a stronger tendency to seek formal psychological support [57]. These findings highlight the need to consider demographic factors in DMHIs, as age and gender influence symptom trajectories and treatment response.

At the start of care, patients using Rula Health’s platform are matched with providers based on stated preferences, including race and ethnicity. This approach reduces patient burden in navigating the provider search process and improves access to culturally responsive care. Observed racial and ethnic differences in symptom trajectories suggest that this MBC-based DMHI may support early symptom reduction among racial and ethnic minority populations, who have historically faced structural barriers to accessing mental health services [58-60]. Black/African American, American Indian/Alaska Native, Native Hawaiian/Pacific Island, and mixed-race individuals experienced faster symptom improvements, particularly within the first 5 visits. The rapid improvements observed in these groups reflect Rula Health’s commitment to providing accessible, high-quality care that meets the needs of diverse patients. Minority groups often enter treatment with greater distress due to systemic barriers to care, which may contribute to a steeper initial recovery trajectory once treatment begins [58-60]. We also found slower symptom reductions among Asian and non-Hispanic individuals, which may be influenced by cultural differences in symptom expression, help-seeking behaviors, or engagement with therapy, as some populations experience higher mental health stigma or prefer alternative coping strategies [61-63]. Future research should also explore how DMHIs, like Rula Health, can further tailor treatment approaches to ensure equitable outcomes across all racial and ethnic backgrounds.

Symptom improvements were most pronounced within their respective diagnostic categories, which aligns with the MBC model leveraged by this DMHI (Rula Health). Individuals with a primary depressive disorder experienced the fastest reduction in depression symptoms, while those with a primary anxiety disorder saw the quickest alleviation of anxiety symptoms. Unlike traditional therapy models, Rula Health’s MBC model ensures ongoing symptom tracking, supporting therapists to adapt treatment to shifting symptom trajectories. Most commercial DMHIs that integrate MBC typically track symptoms every 3 weeks or monthly—less frequently than Rula Health’s approach [42,64]. The MBC model used by Rula Health enables a responsive, data-driven approach that systematically tracks patient-reported outcomes to inform clinical decision-making. This framework facilitates early symptom detection and supports continued improvement across diagnostic categories over the course of up to 12 visits. Later in treatment, individuals with a primary diagnosis of depression improved faster in both depression and anxiety symptoms. Disorder-specific treatments may yield the fastest results in their target symptoms, while cross-diagnostic benefits, even in conditions that tend to be highly comorbid, such as anxiety, depressive, and trauma-related disorders, emerge more gradually. This slower cross-diagnostic improvement may reflect the additional cognitive and emotional processing required to generalize therapeutic gains across disorders [65,66]. Clinically, these results emphasize the importance of maintaining treatment beyond initial symptom relief to maximize broad-spectrum recovery [45,67], an approach that, to our knowledge, is more consistently implemented by Rula Health than by other commercial DMHIs.

### Strengths and Limitations

This study has several strengths. First, it uses a large and diverse sample of patients receiving care through Rula Health’s MBC DMHI, allowing for insights that are generalizable across various demographic and diagnostic groups. Unlike studies relying on highly controlled clinical trials, this research examines real-world clinical data, making the findings more applicable to actual mental health care settings. The longitudinal tracking of symptom changes over 12 sessions provides a detailed picture of how depression and anxiety symptoms evolve over time, offering a nuanced understanding of both rapid early improvements and slower later-stage progress. By identifying key moderators of symptom improvement, such as baseline severity, demographics, and primary diagnoses, it contributes to a more nuanced understanding of variability in treatment response in DMHIs. This study adds to the body of research on DMHIs by investigating how depression and anxiety symptoms evolve over time in Rula Health, an MBC-integrated DMHI, emphasizing the influence of demographic and clinical factors on treatment response.

There are limitations that should be acknowledged. As an observational, non-randomized study, causal conclusions about treatment effectiveness are limited, and future research should incorporate randomized controlled trials to better assess treatment efficacy. Additionally, treatment engagement metrics (such as adherence to sessions, completion rates, and active participation) were not measured, which limited our ability to assess why symptoms improved over time. This study also lacks other variables that could have impacted treatment, such as social support, therapy modality (eg, cognitive behavioral therapy vs acceptance and commitment therapy), comorbid diagnoses, or life stressors. Future research should include these factors to better elucidate the mechanisms underlying treatment response and address these important knowledge gaps. Lastly, post-treatment follow-up data were not collected, preventing the evaluation of whether symptom improvements were maintained after care ended. Future studies should incorporate longitudinal follow-up to assess the durability of treatment effects over time.

### Conclusions

This study provides valuable insights into how depression and anxiety symptoms evolve over time in patients receiving care through Rula Health, an MBC-integrated DMHI. We found that attending more therapy sessions was associated with greater symptom improvement. Symptom trajectories revealed that the most rapid improvements occurred within the first 5 visits, followed by a more gradual decline from visits 6‐12. Baseline severity, demographics, and primary diagnosis also influenced symptom trajectories, highlighting the various factors that shape treatment outcomes in mental health care. Our study highlights the impact of MBC DMHIs on clinical outcomes, providing evidence for the effectiveness of a digitally delivered MBC model in a large real-world patient sample.

## Supplementary material

10.2196/75750 Multimedia Appendix 1 Supplementary material.

## Abbreviations:

DMHI: digital mental health intervention
MBC: measurement-based care
PHQ-9: Patient Health Questionnaire-9
GAD-7: Generalized Anxiety Disorder-7
