# Supplementary material for: Predictors and Correlates of Depression and Anxiety Symptom Trajectories in a Large Digital Mental Health Provider: Retrospective Analysis of Data From Rula Health
Source: J Med Internet Res. 2025 Jul 25;27:e75750. doi: 10.2196/75750 (PMC12296207; doi:10.2196/75750)
Supplement: Multimedia Appendix 1 [file jmir-v27-e75750-s001.docx]

Predictors and correlates of depression and anxiety symptom trajectories in a large digital mental health provider: a retrospective analysis of data from Rula Health

**Supplemental Materials**

**Multiple Imputation for Depression and Anxiety Change Scores and Effect Sizes**

*Methods*

Most patients (79.88% for PHQ-9; 80.00% for GAD-7) had a missing data point at exactly 12 visits. Independent sample t-tests were conducted to assess differences in PHQ-9 and GAD-7 change scores between patients with and without available data at visit 12. Significant differences were observed for both PHQ-9 (t(43,509) = 11.80, p <0.0001, d= 0.46) and GAD-7 (t(44,018) = 10.63, p<0.0001, d=0.37). Given these differences, multiple imputation was applied to estimate missing PHQ-9 and GAD-7 scores at visit 12 (Rubin, 1987) for change score and effect size estimation. One hundred datasets were imputed given our large dataset and high percent of missingness. Predictive mean matching was used to calculate the imputed PHQ-9 and GAD-7 scores. The imputation model included baseline PHQ-9 and GAD-7, last available PHQ-9 and GAD-7, last visit attended with data, age, gender, and diagnosis. Race and ethnicity were left out of the imputation model due to the high missingness in these variables themselves.

A pooled intercept-only regression model was used to obtain the pooled mean change for PHQ-9 and GAD-7 (i.e., to obtain the change effect with no additional predictors). Regression analyses were conducted within each imputed dataset, and results were pooled (Rubin, 2018). Pooled estimates of the mean change, standard errors, and significance values are reported. Pooled standard deviations were also calculated using Rubin’s rules to calculate Cohen’s d with the pooled change score from the regression.

*Results*

**Table S1** shows the results of the pooled intercept-only models predicting PHQ-9 and GAD-7 change scores. Change effects obtained from the pooled regression models were significant for both PHQ-9 (b=-3.21, SE=0.024, *P*<.001) and GAD-7 (b=-3.11, SE 0.020, *P*<.001). Effect sizes were moderate (d_PHQ-9_=-0.53, d_GAD-7_=-0.58). Estimated change scores and effect sizes were similar to those found in the original analysis conducted using last observation carried forward.

| Table S1. Results of the pooled intercept-only regression models for PHQ-9 (N=198,812) and GAD-7 (N=201,081) | | | | |
| --- | --- | --- | --- | --- |
|  | Pooled Mean Change | SE | *P* | Cohen’s d of pooled mean change |
| PHQ | -3.21 | 0.024 | <.001 | -0.53 |
| GAD | -3.11 | 0.020 | <.001 | -0.58 |

*References*

Rubin, D. B. (1987). Multiple imputation for nonresponse in surveys. Wiley.

Rubin, D. B. (2018). Multiple imputation. In *Flexible Imputation of Missing Data, Second Edition* (2nd ed.). Chapman and Hall/CRC.
